# Segregation of Benzoic Acid in Polymer Crystalline Cavities

**DOI:** 10.3390/polym15010177

**Published:** 2022-12-30

**Authors:** Antonietta Cozzolino, Guglielmo Monaco, Paola Rizzo, Gaetano Guerra

**Affiliations:** Dipartimento di Chimica e Biologia “A. Zambelli”, INSTM Research Unit, Università degli Studi di Salerno, Via Giovanni Paolo II, 132-84084 Fisciano, Italy; acozzolino@unisa.it (A.C.); gmonaco@unisa.it (G.M.)

**Keywords:** nanoporous-crystalline forms, crystalline polymer hosts, benzoic acid guest molecules, polarized FTIR, WAXD

## Abstract

Benzoic acid (BA) and its derivatives are very attractive because of their pharmacological properties, such as antioxidant, radical-regulating, antiviral, antitumor, anti-inflammatory, antimicrobial and antifungal. Syndiotactic polystyrene (sPS) and poly(2,6-dimethyl-1,4-phenylene)oxide (PPO) films exhibiting co-crystalline phases with BA were prepared and characterized by WAXD, FTIR and polarized FTIR measurements. The FTIR measurements clearly showed that BA was present mainly as a dimer in the crystalline channels of the ε form of sPS as well as in the α form of PPO, as generally occurs not only in the solid state but also in organic dilute solutions. BA was instead present as isolated molecules in the crystalline cavities of the δ form of sPS. In fact, the FTIR spectra of BA guest molecules exhibited vibrational peaks close to those of BA in its vapor phase. Hence, the nanoporous-crystalline δ form of sPS not only avoids additive aggregation but also leads to the separation of dimeric molecules and the segregation of monomeric BA.

## 1. Introduction

In the last two decades, a new route for the inclusion of functional additives in polymer samples, which leads to the prevailing location of the active molecules in the polymer crystalline phases rather than in the polymer amorphous phases, was revealed [1,2]. These studies refer to two commercial polymers, syndiotactic polystyrene (sPS) [3,4,5] and poly(2,6-dimethyl-1,4-phenylne)oxide (PPO) [6,7,8], which exhibit nanoporous-crystalline (NC) phases. The main feature of these NC phases, named δ [1,3] and ε [1,5] for the sPS and α and β for PPO [6], is their density, which is lower than for corresponding amorphous phases. Active guest molecules can be absorbed in the crystalline cavities of the sPS δ phase or in the crystalline channels of the sPS ε phase or of the PPO α and β phases, thus leading to corresponding co-crystalline (CC) phases [9,10,11,12,13,14,15,16]. The inclusion of functional molecules in crystalline phases avoids their aggregations which, in general, could be detrimental for the film and fiber’s physical properties.

In particular, functional films based on the CC phases of these two polymers, with the guests being chromophore [9,10], photoreactive [12,13], chiraloptical [14] or antimicrobial (e.g., with carvacrol [15] and hexanal [16]), were described.

Two recent papers by our group describe the CC phases of sPS with aliphatic carboxylic acids, showing that the crystalline channels of the ε form were able to host long carboxylic acids as well as dicarboxylic acids [17,18]. This selective and fast uptake of carboxylic acids in the crystalline channels of the sPS ε form is due to the formation of hydrogen bonded linear dimers and linear polymers for the monocarboxylic and dicarboxylic acid guests, respectively [17,18]. Moreover, the inclusion of hexanoic acid as an isolated guest of the crystalline cavities of the sPS δ form was also described [17].

Benzoic acid (BA) and its derivatives are very attractive because of their pharmacological properties, such as antioxidant, radical-regulating, antiviral, antitumor, anti-inflammatory, antimicrobial and antifungal [19,20,21]. These low toxicity compounds are widely used in medicine and can be found in various cosmetic products, medications and food as additives and preservatives to certain approved concentrations [22,23]. In fact, many studies report on the release of BA and derivatives [24,25,26,27,28,29].

In this paper, we characterized films of sPS and PPO including BA molecules in their amorphous and crystalline phases. In particular, axially oriented sPS and PPO films [30] were used to obtain information on the crystalline structure by WAXD fiber patterns, as well as information on the aggregation and orientation of the guest molecules in the polymeric amorphous and crystalline phases, by polarized FTIR measurements. The present study shows that the NC δ form of sPS not only avoids additive aggregation but also leads to the separation of BA dimers and, hence, the appearance of vibrational peaks typical of vapor phase molecules.

## 2. Experimental Section

### 2.1. Materials

Benzoic acid (BA) is a carboxylic acid with T_m_ = 122 °C and T_b_ = 249 °C, showing an acid dissociation constant p*K*_a_ = 4.2 at 25 °C. The BA and all used solvents were provided by Sigma-Aldrich (St. Louis, MO, USA) and used without additional purification.

The syndiotactic polystyrene was supplied from Idemitsu (Xarec 90ZC) (Tokyo, Japan) and had a content of syndiotactic triads higher than 98%; its molar weight was M_w_ = 140 kg·mol^−1^ (polydispersity M_w_/M_n_ = 2.0). Amorphous sPS films were obtained through a blown extrusion process using a melt temperature of 290 °C with a blow-up ratio of 2.5 and a draw ratio of 8.

The amorphous sPS films were stretched up to a draw ratio of 4.0 using an elongation rate of 10 mm/min with a dynamometer INSTRON 4301 (Norwood, MA, USA) at 105 °C. The axial stretching induces the formation of a highly oriented mesomorphic phase, which exhibits zig-zag planar chains [31].

Axially oriented sPS films with the NC δ form were obtained by immersion of axially stretched mesomorphic films in dichloromethane at room temperature for 1 night, followed by the sorption of volatile guest molecules (acetonitrile) for 3 h for guest removal. Axially oriented γ form sPS films were obtained by annealing δ form films at 170 °C for 1 h. Axially oriented NC ε form sPS films were obtained by immersion of the axially oriented γ form film in chloroform for 1 h, followed by chloroform removal by acetonitrile sorption for 3 h. The thicknesses of the axially stretched mesomorphic and NC films were in the range 60–80 μm.

Poly(2,6-dimethyl 1,4-phenylene)oxide (PPO), with a weight-average molecular mass (M_w_) of 350 kg·mol^−1^ is from SABIC. Films exhibiting axial orientation of α NC phases were obtained by immersion of axially stretched amorphous films in limonene at room temperature for 1 night, followed by limonene removal by acetonitrile sorption for 3 h. Film stretching experiments were conducted by a dynamometer INSTRON 4301, at 220 °C, up to a draw ratio of 4.0 by using elongation rate of 10 mm/min. The thickness of the axially oriented NC α form films is close to 140 μm.

Axially oriented CC films were obtained by immersion in a 30 wt% BA/acetone solution at room temperature for different times (2 h, 3 h, and overnight), followed by acetone desorption at 60 °C from 2 to 10 days.

### 2.2. Techniques and Methods

Wide-angle X-ray diffraction (WAXD) patterns were collected using a Bruker automatic diffractometer (Billerica, MA, USA), with CuKα radiation.

Fourier transform infrared (FTIR) spectra were collected using a Vertex 70 Bruker spectrometer in the wavenumber range 4000–400 cm^−1^ and with a resolution of 2.0 cm^−1^. The Bruker spectrometer (Billerica, MA, USA) was equipped with a deuterated triglycine sulphate (DTGS) detector and a Ge/KBr beam splitter, and the frequency scale was internally calibrated to 0.01 cm^−1^ using a He−Ne laser. Polarized infrared spectra were obtained using a SPECAC 12000 wire grid polarizer. In order to reduce the noise, 32 scans were signal averaged.

The degree of crystallinity (*X_c_*) of the films was performed by a FTIR spectral subtraction procedure following the formula K = (l/l’)(1 − *X_c_*), where K is the subtraction coefficient, l and l’ are the thickness of the sample and of an amorphous reference film. The ratio l/l’ was spectroscopically estimated from the absorbance ratio of a conformationally insensitive peak (at 1601 cm^−1^ for sPS and 960 cm^−1^ for PPO). The degree of crystallinity evaluated for all semicrystalline films was close to 25–30%.

The guest content in all the films was evaluated by the intensity of the FTIR peaks, calibrated using thermogravimetric (TGA) measurements. The TGA measurements were performed at a scan rate of 10 °C/min and in the temperature range 25–350 °C, using a TG 209 F1 *Netzsch* (Oberfranken, Bavaria, Germany).

The order parameter of the polymer crystalline phases (*S_p_*) was calculated with the formula:

(1) $$S_{p}=\frac{R_{p}-1}{R_{p}+2}$$

where *R* = A_//_/A_⊥_ is the dichroic ratio, and A_//_ and A_⊥_ are the absorbance intensities mea-sured with polarization plane parallel and perpendicular to the draw direction, respectively. This orientation factor is equal to zero for random crystallite orientation, while it is equal to +1 and −0.5 for the orientation of all polymer chain axes of the crystallites, being parallel and perpendicular to the stretching direction, respectively.

The polarized FTIR spectra of the CC films also allowed for an analogous evaluation of the order parameter of BA monomeric and dimeric guest molecules (*S_m_* and *S_d_*, respectively), with respect to the film stretching direction:

(2) $$S_{m}=\frac{R_{m}-1}{R_{m}+2}\;\;\;;\;\;\;\;\;S_{d}=\frac{R_{d}-1}{R_{d}+2}$$

where *R_m_* and *R_d_* are the dichroic ratios, as evaluated for monomeric and dimeric vibrational peaks.

The FTIR spectra were fitted as sums of Lorentzian peaks, as described in ref. [32]. In order to obtain the percentage of monomers and dimers, integrated areas obtained by the fitting were corrected by molar extinction coefficients estimated by a quantum mechanical computation on monomers and dimers in the gas phase, in the harmonic approximation at the APFD [33]/6-311+G** level, using Gaussian16 [34].

## 3. Results and Discussion

### 3.1. Co-Crystalline Phases sPS/BA

The sorption of BA in axially oriented sPS films was conducted at room temperature, using concentrated acetone solutions (30wt% of BA). The BA guest uptake after 14 h of room temperature treatment was low for the films exhibiting the dense γ phase (lower than 1 wt%), while it was much higher for films that were zig-zag planar mesomorphic (axially stretched amorphous) or exhibiting the NC δ and NC ε phases (4.1 wt%, 5.1 wt% and 6.8 wt%, respectively).

**Figure 1:**
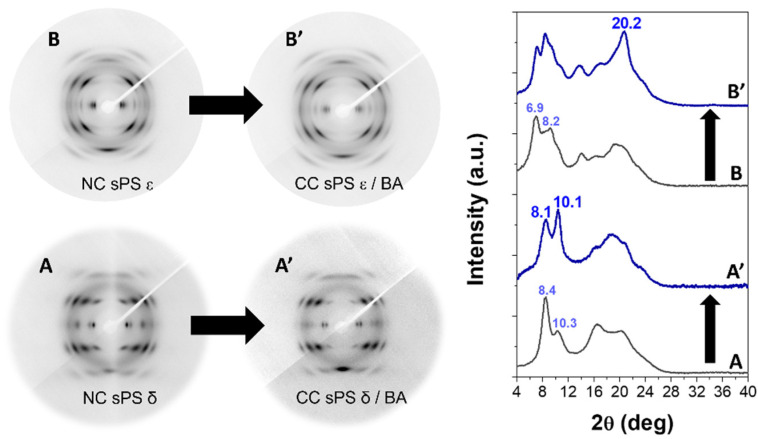
Two-dimensional WAXD fiber patterns and related equatorial intensity profiles of sPS films presenting nanoporous crystalline (NC) and co-crystalline (CC) δ (**A**,**A’**) and ε (**B**,**B’**) phases. Diffraction angles of relevant reflections of NC and CC phases are indicated.

The WAXD fiber patterns, taken with a two-dimensional diffractometer (on the left) and corresponding equatorial diffraction profiles (on the right) for sPS axially stretched films, exhibiting the NC δ and ε phases, before and after sorption of BA, are shown in Figure 1. The WAXD patterns of the NC δ film (Figure 1A) presented 010 and $\bar{2}10$ reflections (at 2θ = 8.4° and 10.3°, respectively) typical of the NC monoclinic δ phase [1]; after sorption of BA, the WAXD patterns showed shifts of 010 and $\bar{2}10$ diffraction peaks to 2θ = 8.1° and 10.1° (Figure 1A’), as well as a reduced intensity of the 010 peak, which are typical of CC monoclinic δ phases [1].

The WAXD patterns of the NC ε film (Figure 1B) presented 110 and 020 reflections (at 2θ = 6.9° and 8.2°, respectively) typical of the NC orthorhombic ε phase [1]; after sorption of the BA, the intensity of these equatorial peaks markedly decreased with respect to the intensity of the first-layer line reflections (mainly at 2θ = 20.2°), as is typical of CC ε phases [1].

**Figure 2:**
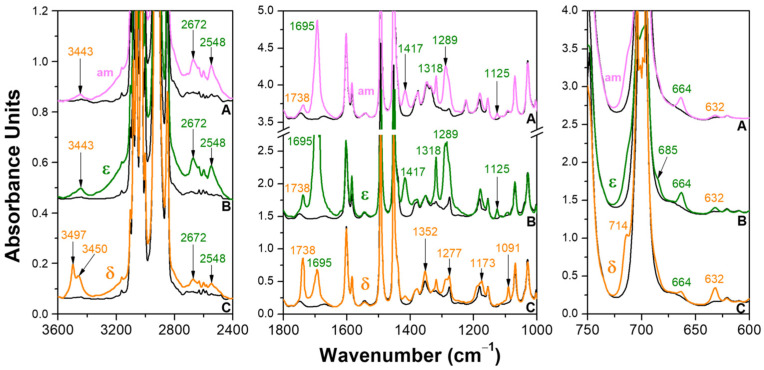
FTIR spectra, for three spectral ranges (3600–3200 cm^−1^, 1800–1000 cm^−1^ and 750–600 cm^−1^) of axially oriented sPS films before (black lines) and after BA sorption (colored lines): (**A**) zig-zag planar mesomorphic; (**B**) NC ε form; (**C**) NC δ form. Peaks due to the isolated and dimeric BA molecules are labeled by orange and green numbers, respectively.

The FTIR spectra of the axially oriented sPS films that were zig-zag planar mesomorphic or exhibiting the NC δ and ε phases, before (black lines) and after 14 h of BA sorption (colored lines), are shown in Figure 2. The FTIR peaks of the BA guest of the zig-zag planar mesomorphic film (Figure 2A) and of the CC sPS/BA ε form film (Figure 2B) were similar and essentially corresponded to the peaks of the BA dimer (labeled by the green numbers in Figure 2). These peak positions are listed in the 3rd column of Table 1 and are compared with the list of the BA dimer peaks, as observed for solid BA, from KBr pellets (1st column in Table 1) [35] as well as from mull (2nd column of Table 1) [36]. In the spectra of both the mesomorphic and CC ε films, in addition to the vibrational peaks of the BA dimer, additional weak peaks (labeled with orange numbers in Figure 2, at 3443, 1738 and 632 cm^−1^) were shown, which can be attributed to isolated BA molecules. Quantitative evaluations were conducted considering the pair of FTIR peaks at 1738/1695 cm^−1^ for the mesomorphic film and at 663/632 cm^−1^ for the CC ε film, as described in the experimental section and in the supporting section (Table S1, Figures S2 and S3), indicate that roughly 10% and 15% of BA was present as isolated molecules, for the mesomorphic film (and, hence, for its amorphous phase) and for the CC ε film, respectively. This result is not surprising, because BA is present essentially only as a dimer, even in dilute organic solutions [36].

A completely different behavior was observed for the FTIR spectra of the CC sPS/BA δ form films (Figure 2C). In fact, the main BA peaks were of isolated BA (monomer) and are indicated by the orange numbers in Figure 2. These peaks are listed in the third column of Table 2 and compared with the list of the peaks of BA monomer as obtained in Ar matrices (1st column in Table 2) [37] as well as for the high-temperature vapor of BA (2nd column of Table 2) [36]. In addition to the vibrational peaks of BA monomer, additional peaks (labeled with green numbers in Figure 2, at 2672, 2548, 1695 and 664 cm^−1^) were present, which can be attributed to the BA dimer. Quantitative evaluations considering the pair of FTIR peaks at 1738/1695 cm^−1^ (see Table S1 and Figure S1 in Supporting section) indicate that nearly 50% of the BA molecules were present as isolated guest molecules for the CC δ film.

**Figure 3:**
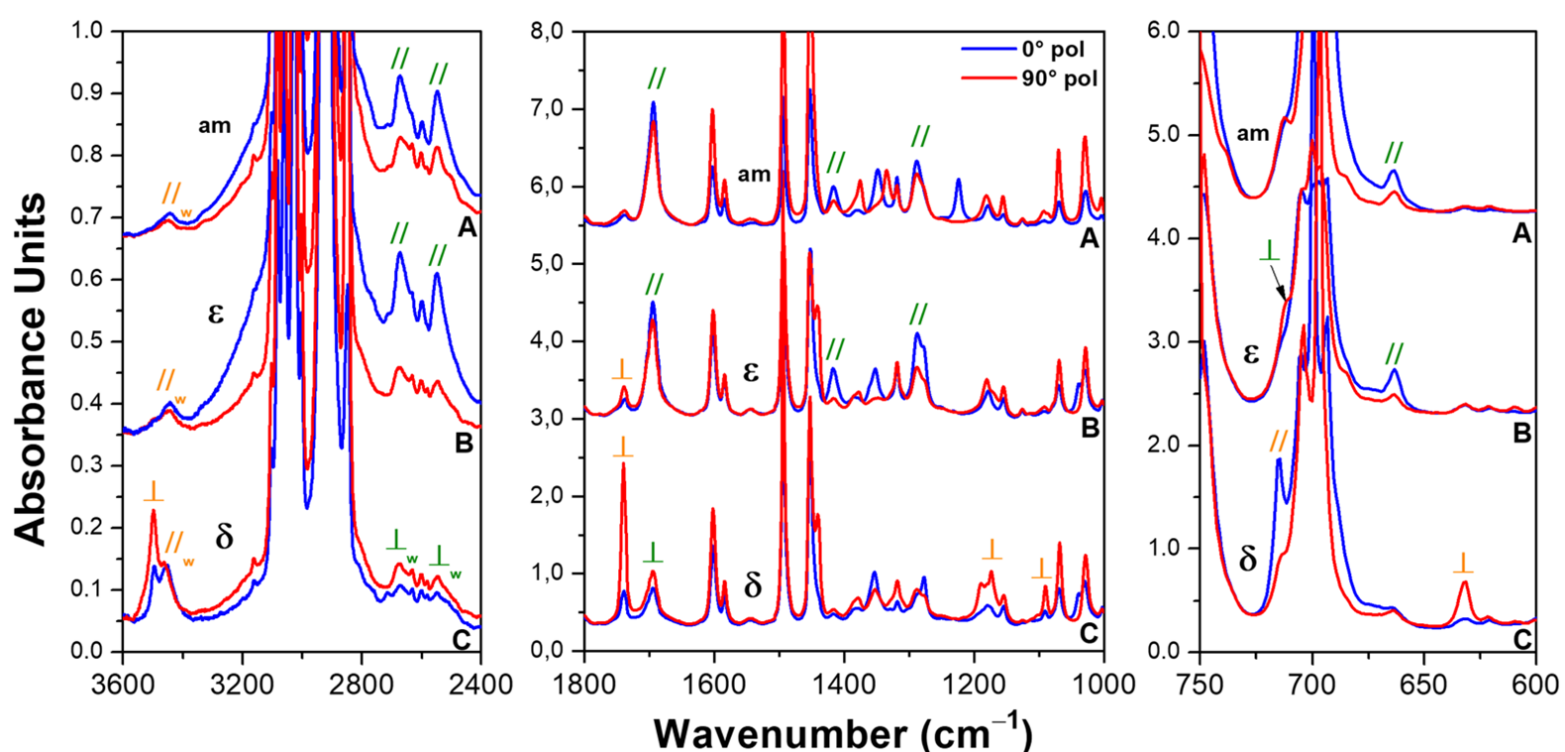
Polarized FTIR spectra measured with the polarization plane parallel (blue lines) and perpendicular (red lines) to the film stretching direction, in three spectral ranges (3600–2400, 1800–1000 and 750–600 cm^−1^), of axially oriented sPS films: (**A**) zig-zag planar mesomorphic; (**B**) CC ε form; (**C**) CC δ form. The labels // and ⊥ indicate that the guest peak absorbance was higher for the spectra with the polarization parallel and perpendicular to the film stretching direction, respectively. The green and orange labels indicate the peaks corresponding to the dimeric and isolated BA, respectively.

The linear dichroism of the dimeric and monomeric guest peaks of Table 1 and Table 2, respectively, was studied using the polarized FTIR spectra of the axially oriented mesomorphic and CC (δ or ε) sPS films (Figure 3). The parallelism of the zig-zag planar chains of the dense mesomorphic phase with respect to the stretching direction was nearly complete (orientation factor, as evaluated from the 1222 cm^−1^ peak *S_p,meso_* > 0.95). The orientation factor of the host helical crystalline polymer phases, exhibiting s(2/1)2 helices, was also high and was evaluated based on the polymer host peak at 572 cm^−1^: *S_p,__δ_* = 0.85 and *S_p,ε_* = 0.80.

The polarized spectra in Figure 3 show that many guest peaks were dichroic, not only for the CC films that absorb BA molecules both in their amorphous and crystalline phases but also for the mesomorphic film that absorbs BA molecules only in its amorphous phases. In particular, the labels // and ⊥ indicate that the peak absorbance was higher for the spectra with the polarization parallel or perpendicular to the film stretching direction, respectively. These // and ⊥ labels are colored in green and orange when the guest vibrational peak was of dimeric and of isolated BA molecules, respectively. This information relative to the dichroism of the peaks of the dimeric and isolated BA is also included in Table 1 and Table 2, respectively.

For the polarized spectra of the CC δ form film (Figure 3C), the peaks of isolated BA molecules exhibited very intense dichroism. For instance, the orientation factor of the peaks of the monomeric guest was *S_m,1738,__δ_* = −0.36, *S_m,1090,__δ_* = −0.33 and *S_m,632,__δ_* = −0.36, i.e., not far from the limit value of −0.5, corresponding to a perfect orientation of all BA guest molecules. Particularly informative was the carbonyl region, where the sign of the dichroism (⊥) of the carbonyl stretching of the isolated molecule (at 1738 cm^−1^) indicated that the BA guest molecules of the δ form were preferentially perpendicular to the stretching direction, as already observed for many aromatic guest molecules of the δ form of sPS [1,38]. In addition, the region of the O-H stretching of isolated BA molecules in the CC δ form film was also interesting (Figure 3C). In fact, in addition to a poorly dichroic peak at 3450 cm^−1^ corresponding to isolated BA molecules in the amorphous sPS phases, a highly dichroic peak at 3497 cm^−1^ corresponding to isolated BA molecules in the cavities of the δ form was also observed.

For the polarized spectra of the mesomorphic film (Figure 3A) and of the CC ε film (Figure 3B), the kind of dichroism (// or ⊥) was the same for all the observed peaks. The dichroism of the largely prevailing BA dimers was opposite with respect to those observed for the minor content of BA dimers included in the δ form film (e.g., dichroism of the peaks at 2672, 2548 and 1695 cm^−1^). This indicates that, both in the oriented amorphous phase as well as in the crystalline channels of the CC ε phase, the phenyl rings of the BA dimers were preferentially parallel to the film stretching direction. This can be easily rationalized by the expected preferential axial orientation of the long axes of the hydrogen bonded BA dimers. Moreover, the higher dichroism intensity for the CC ε film (*S_d,2672,am_* = +0.36; *S_d,2672,ε_* = +0.55; *S_d,1417,am_* = +0.23; *S_d,1417,ε_* = +0.56; *S_d,664,am_* = +0.29; *S_d,664,ε_* = +0.40) clearly indicates the occurrence of a higher degree of orientation of the dimeric guest molecules in the channels of the CC ε phase.

It is worth adding that the sign of the dichroism of the 1738 cm^−1^ peak was the same for the CC ε and CC δ films (*S_m,1738,ε_* = −0.17, *S_m,1738,__δ_* = −0.36). This indicates that, as well as for both the CC δ and CC ε phases, the orientation of the isolated BA guest plane was preferentially perpendicular to the stretching direction.

**Figure 4:**
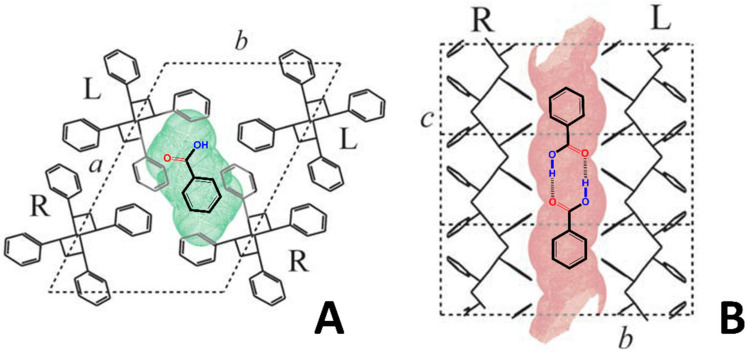
Schematic views of the preferential aggregation and orientation of BA guest molecules in the CC phases of sPS: (**A**) *ab* is the projection of the CC δ form with isolated BA molecules and guest molecular plane preferentially perpendicular to the helical chain axes; (**B**) *bc* is the projection of the CC ε phase with dimeric BA molecules and guest molecular planes preferentially parallel to the helical chain axes.

To summarize, the FTIR spectra of Figure 2 and Figure 3 show that the BA molecules were prevailingly present as dimers, preferentially parallel to the film stretching direction, both in the amorphous and CC ε phases of sPS. The BA molecules, on the contrary, were prevailingly present as isolated molecules and preferentially perpendicular to the film stretching direction in the CC δ phases of sPS. A schematic representation of the BA guest molecules as monomer in the crystalline cavities of the δ form and as dimer in the crystalline channels of the ε form is reported in Figure 4A,B, respectively.

### 3.2. Co-Crystalline Phases PPO/BA

**Figure 5:**
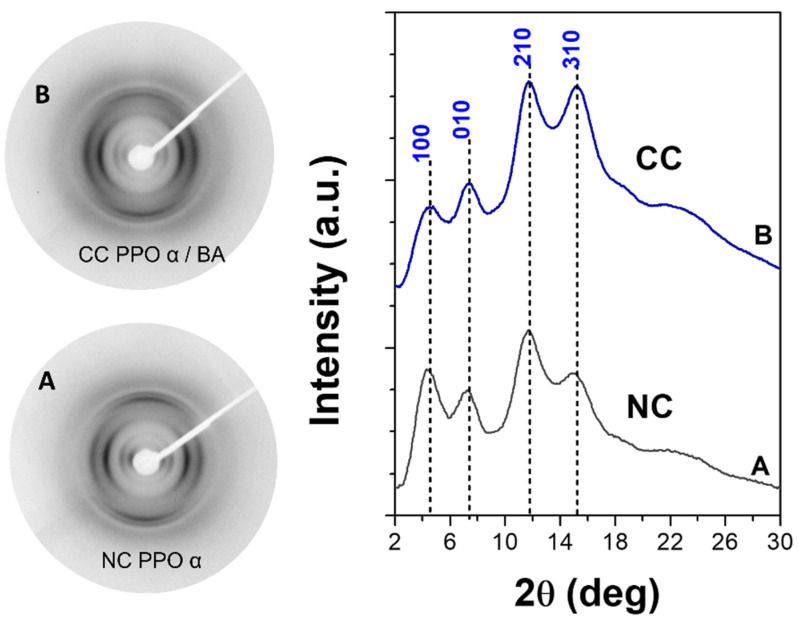
Two-dimensional wide-angle X-ray diffraction patterns and related equatorial intensity profiles of axially oriented PPO films presenting the (**A**) NC α form and (**B**) CC α form with BA. Miller indexes of relevant equatorial reflections of the CC phase are indicated.

The WAXD fiber patterns, taken with a two-dimensional diffractometer (on the left) and corresponding equatorial diffraction profiles (on the right) for a PPO axially stretched film, exhibiting the NC α phase, before and after sorption of BA (8 wt%, from 30 wt% BA/acetone solution for 2 h and after subsequent acetone desorption), are shown in Figure 5. The WAXD patterns of the NC α film (Figure 5A) presented typical diffraction peaks at 2θ_CuKα_ ≈ 4.5°, 7.1°, 11.3° and 15.0°, corresponding to the (100), (010), (210) and (310) crystal planes. After the sorption of the BA molecules, the pattern showed a reduced intensity of the 100 and 010 reflections as well as an increased intensity of the 310 reflection, clearly indicating the formation of the α CC phase (e.g., Figure 2 in [6]).

**Figure 6:**
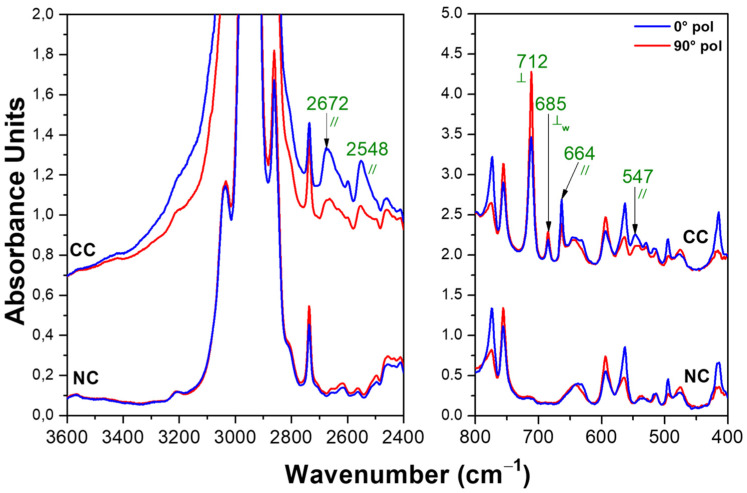
Polarized FTIR spectra measured with the polarization plane parallel (blue lines) and perpendicular (red lines) to the film stretching direction for two spectral ranges (3600–2400 and 800–400 cm^−1^) for an axially oriented PPO film exhibiting the α NC phase, before and after BA sorption, leading to the corresponding CC phase. explanation of labels // and ⊥ can be found in Figure 3. The green labels indicate the peaks.

The polarized FTIR spectra of the axially oriented α PPO films before and after 8 wt% of BA uptake are shown in Figure 6. In agreement with the literature [30], the FTIR peaks (at 773, 756, 495 and 414 cm^−1^) typical of the α NC host phase were dichroic (e.g., *S_p,495_* = +0.33).

The positions of the guest peaks as well as their possible dichroism (⊥ or //) are listed in the 4th column of Table 1. It is worth noting that, in the accessible spectral regions with low absorbance of the host polymer, the positions and dichroism of all guest peaks were coincident with those of BA dimers, being a guest of the sPS ε form film (3rd column of Table 1).

It is also worth noting the absence of FTIR peaks (e.g., at nearly 3500 cm^−1^) of the isolated BA guest molecules, which (although weak) occurred for the BA molecules in the amorphous and CC ε phases of sPS. This indicated that both in the high free-volume amorphous phase of PPO as well as in the large intrahelical channels of the α form of the PPO [2], BA molecules essentially were only present as dimers.

These data also confirm that the empty spaces in the NC form of PPO were distributed as channels (as occurred for the ε phase of sPS) rather than as isolated cavities (as occurred for the δ phase of sPS).

## 4. Conclusions

The sorption of BA molecules in films presenting NC δ and ε phases for the sPS and NC α phase for PPO were studied. The formation of δ and ε sPS/BA CC phases as well as α PPO/BA CC phases were shown by typical changes in the WAXD fiber patterns.

The polarized FTIR spectra showed that, in the amorphous and CC ε phases of sPS, the BA molecules were prevailingly present as dimers, and their molecular planes were preferentially parallel to the film stretching direction. In the CC δ phases of sPS, on the contrary, the BA molecules were prevailingly present as isolated molecules, and their molecular planes were preferentially perpendicular to the film stretching direction.

The polarized FTIR spectra also showed that, both in the high free-volume amorphous phase of PPO as well as in the large intrahelical channels of the α form of PPO, the BA molecules were essentially present only as dimers. These data also confirm that the empty space in the NC form of PPO was distributed as channels (as occurs for the ε phase of sPS) rather than as isolated cavities (as occurred for the δ phase of sPS).

Hence, the present study shows that only the NC δ form of sPS was able to isolate BA guest molecules, even disrupting their strong intermolecular hydrogen bonds, which are present not only in the BA crystals but also in the diluted solutions [36]. The use of the NC δ form of sPS not only avoids additive aggregation but even leads to the separation of the dimeric molecules. This segregation of the molecules allows for the spectroscopic characterization of the isolated molecules already at room temperature and could be relevant to the control kinetics of the release of active molecules.

## Figures and Tables

**Table 1:** FTIR peaks of BA dimers as observed for solid BA (KBr pellets [35] or mull [36]) and for BA guest molecules in axially stretched ε-form sPS and α-form PPO films.

Solid BA [35]	Solid BA [36] s = Strong m = Medium w = Weak vw = Very Weak	BA in Mesomorphic or in CC ε sPS	BA in CC α PPO
552	552 vw	na	547 //
668	664 vw	664 //	664 //
685	682 vw	685 ⊥	685 ⊥
708	707 s	712 ⊥	712 ⊥
- -	800 vw	797 //	797 //
806	807 vw	808 nd	808 nd
936	932 w	936 nd	936 nd
1026	1023 vw	na	na
1073	1070 vw	na	1070
1129	1125 vw	1125 nd	1125
1181	1184 vw	na	na
1294	1290 m	1289 //	na
1327	1323 m	1318 nd	na
1426	1420 w	1417 //	na
1454	1450 w	na	na
1497	1495 vw	na	na
1584	1580 vw	na	na
1688	1685 s	1695 //	1695 nd
- -	2560 vw	2548 //	2548 //
- -	2603 vw	2600 //	2600 //
- -	2670 vw	2672 //	2672 //
3012	3005 vw	na	na
3073	3078 vw	na	na

na = not accessible; nd = not dichroic; // and ⊥ = dichroic peak with A_//_ > A_⊥_ and A_//_ < A_⊥_, respectively.

**Table 2:** FTIR peaks of isolated BA as taken from [37] (in argon matrix), as observed in the vapor phase spectra (at 433 K) [36] and in the axially stretched δ-form sPS film.

BA Isolated in Argon	BA Vapor s = Strong m = Medium w = Weak vw = Very Weak	BA in CC δ sPS
565	575 vw	na
628	632 vw	632 ⊥
711	715 w	714 //
- -	817 vw	808 nd
937	- -	936 nd
1027	1010 vw	na
1066	- -	na
1086	1084 m	1091 ⊥
1169	- -	1173 ⊥
1185	1184 m	1189 ⊥
1275	1290 vw	1277
1347	1370 vw	1352
1606	1600 vw	na
1752	1765 s	1738 ⊥
3567	3500 vw	3450 //
	3575 m	3497 ⊥

na = not accessible; nd = not dichroic; // and ⊥ = dichroic peak with A_//_ > A_⊥_ and A_//_ < A_⊥_, respectively.

## Data Availability

The data presented in this study are available upon request from the corresponding author.

## Supplementary Materials

The following supporting information can be downloaded at: https://www.mdpi.com/2073-4360/15/1/177/s1, Figure S1: Fit of the infrared spectrum of CC δ sPS/BA film; Figure S2: Fit of the infrared spectrum of CC ε sPS/BA film; Figure S3: Fit of the infrared spectrum of sPS mesoform film; Table S1: Selected outcomes of the quantitative analysis.
