# Supplementary material for: Segregation of Benzoic Acid in Polymer Crystalline Cavities
Source: Polymers (Basel). 2022 Dec 30;15(1):177. doi: 10.3390/polym15010177 (PMC9823889; doi:10.3390/polym15010177)
Supplement: Supplementary file 1 [file polymers-15-00177-s001.zip › polymers-2096451-supplementary-done.pdf]

polymers

MDPI

Article

Segregation of benzoic acid in polymer crystalline cavities

Antonietta Cozzolino, Guglielmo Monaco, Paola Rizzo* and Gaetano Guerra*

Dipartimento di Chimica e Biologia "A. Zambelli" and INSTM Research Unit, Università degli Studi di Salerno, Via Giovanni Paolo II, 13284084 Fisciano, Italy

* Correspondence: prizzo@unisa.it (P.R.); gguerra@unisa.it (G.G.)

SUPPORTING INFORMATION

Fits of infrared spectra

A quantitative evaluation of the relative amount of isolated and hydrogen-bonded guest molecules in all sPS films have been performed. In particular, the molar ratio of acid absorbed as monomer and dimer, $r_{\mathrm{mon}} = \frac{n_{\mathrm{mon}}}{2n_{\mathrm{dim}}}$, has been estimated from the ratio of integrated absorbances of the peaks at 1738 and 1695 cm^{-1} and the DFT-derived ratio of molar extinction, according to the following formula:

$$r_{\mathrm{mon}} = \frac{\frac{1}{2}\frac{\varepsilon_{1695}}{\varepsilon_{1738}}\left(I_{1738}^{h+\mathrm{BA}} - I_{1748}^{h}\frac{I_{1601}^{h+\mathrm{BA}}}{I_{1601}^{h}}\right)}{I_{1695}^{h+\mathrm{BA}}} \quad \text{(S1)}$$

where the superscript h stands for the crystalline host. The molar fraction can be computed as $x_{\mathrm{mon}} = \frac{r_{\mathrm{mon}}}{1+r_{\mathrm{mon}}}$. Equation S1 includes a correction for host absorption, as sPS has a small absorption at around 1748 cm^{-1} which falls under the 1738 cm^{-1} peak of the cocrystals; the correction must cope with the different widths of the films, estimated from the intensity of the conformationally insensitive 1601 cm^{-1} peak.[1]

In the case of the epsilon sample with saturation of the signal at 1695 cm^{-1}, the 664/632 cm^{-1} pair of peaks was used instead. The molar absorption coefficients to be used in Eq. S1 have been estimated by gas-phase DFT computations. The results, gathered in Table S1 show that most of BA is adsorbed as dimer in the epsilon form, while comparable amounts of acid adsorbed as monomer or dimer are found in the delta form.

Citation: Cozzolino, A.; Monaco, G.; Rizzo, P.; Guerra, G. Segregation of Benzoic Acid in Polymer Crystalline Cavities. *Polymers* **2023**, *15*, 177. https://doi.org/10.3390/polym15010177

Academic Editor: Francesco Paolo La Mantia

Received: 29 November 2022
Revised: 15 December 2022
Accepted: 26 December 2022
Published: 30 December 2022

Copyright: © 2022 by the authors. Licensee MDPI, Basel, Switzerland. This article is an open access article distributed under the terms and conditions of the Creative Commons Attribution (CC BY) license (https://creativecommons.org/licenses/by/4.0/).

Table S1: Fitted central wavenumber and integrated areas I (cm^{-1}) of the peaks used to quantify the fraction of acid adsorbed as monomer according to Eq. S1. Computed values, in columns 2 and 3, have been obtained as described in the Experimental Section. Computed frequencies have been scaled by 0.969 to partially compensate for anharmonicity.

Sample	$\tilde{\nu}_c$ (cm^{-1})	ε ($km\ mol^{-1}$)	$\tilde{\nu}$ (cm^{-1})	I (cm^{-1})	r_{mon}	x_{mon}
CC sPS ε/BA	1757.1	396	1739.2	14.85	--	--
	1703.5	1022	1694.0	SAT		
	663.8	39.6	663.6	8.90	0.18	0.15
	620.1	48.2	632.2	4.69		
			1601.9	36.90		
CC sPS δ/BA			1739.4	23.79	1.06	0.51
			1694.7	26.67		
			664.9	1.77	0.67	0.40
			632.2	2.86		
			1601.4	26.64		
sPS mesoform			1740.4	3.10	0.13	0.12
			1694.3	29.64		
			664.1	3.08	0.07	0.07
			631.8	0.52		
NC sPS ε			1748.4	2.78		
			1601.6	15.72		
NC sPS δ			1747.2	3.30		
			1601.3	17.85		

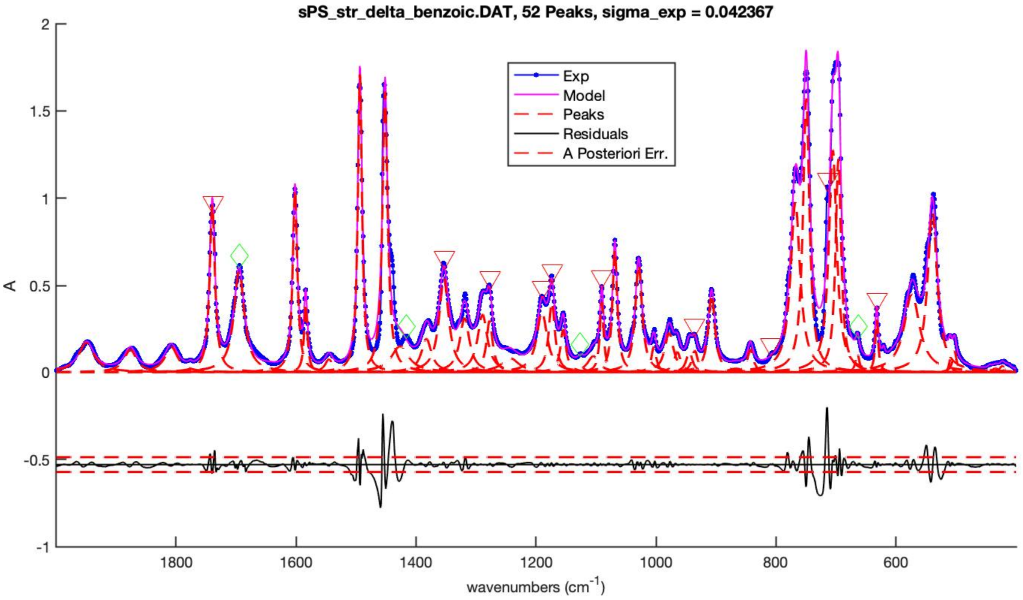

Figure S1. Portion of the spectrum of the CC sPS δ/BA film, together with its fit in terms of sum of Lorentzians, the residuals on the bottom and the error levels. Peaks attributed to the monomer (dimer) of BA are marked with a red triangle (a green diamond).

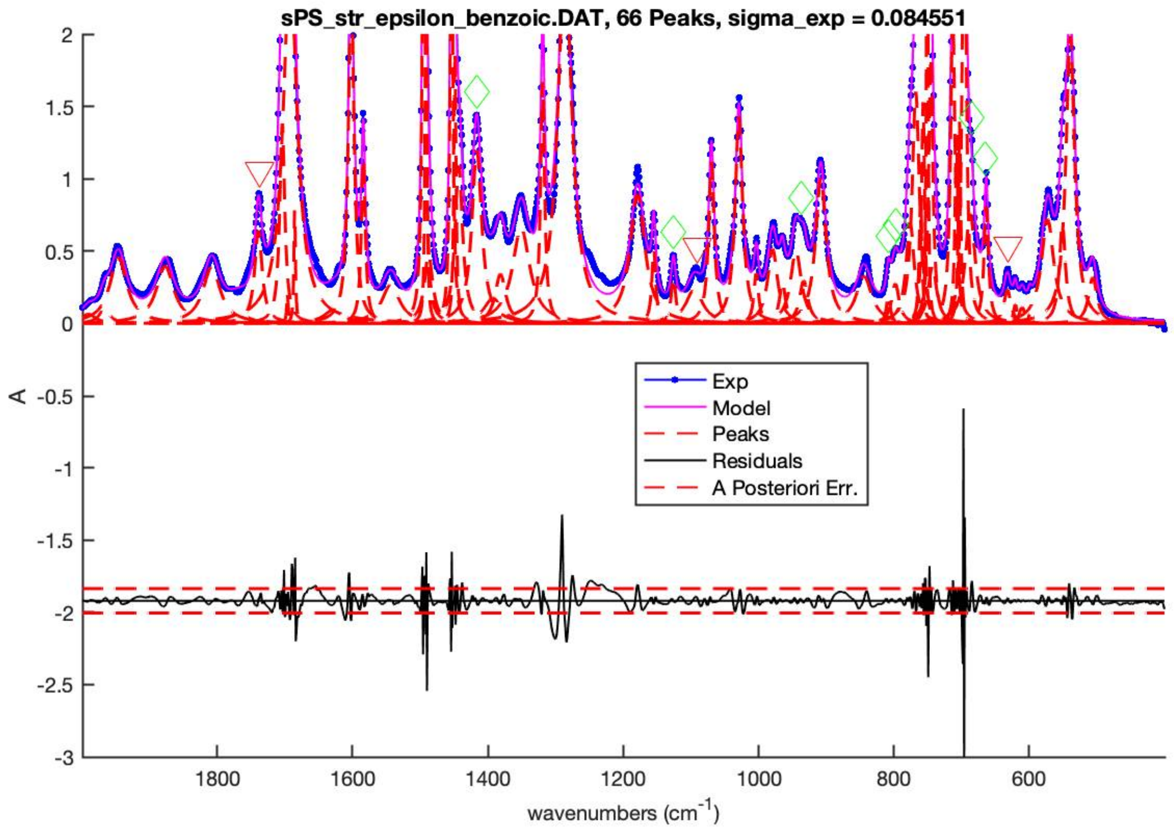

Figure S2. Portion of the spectrum of the CC sPS ε/BA film, together with its fit in terms of sum of Lorentzians, the residuals on the bottom and the error levels. Peaks attributed to the monomer (dimer) of BA are marked with a red triangle (a green diamond).

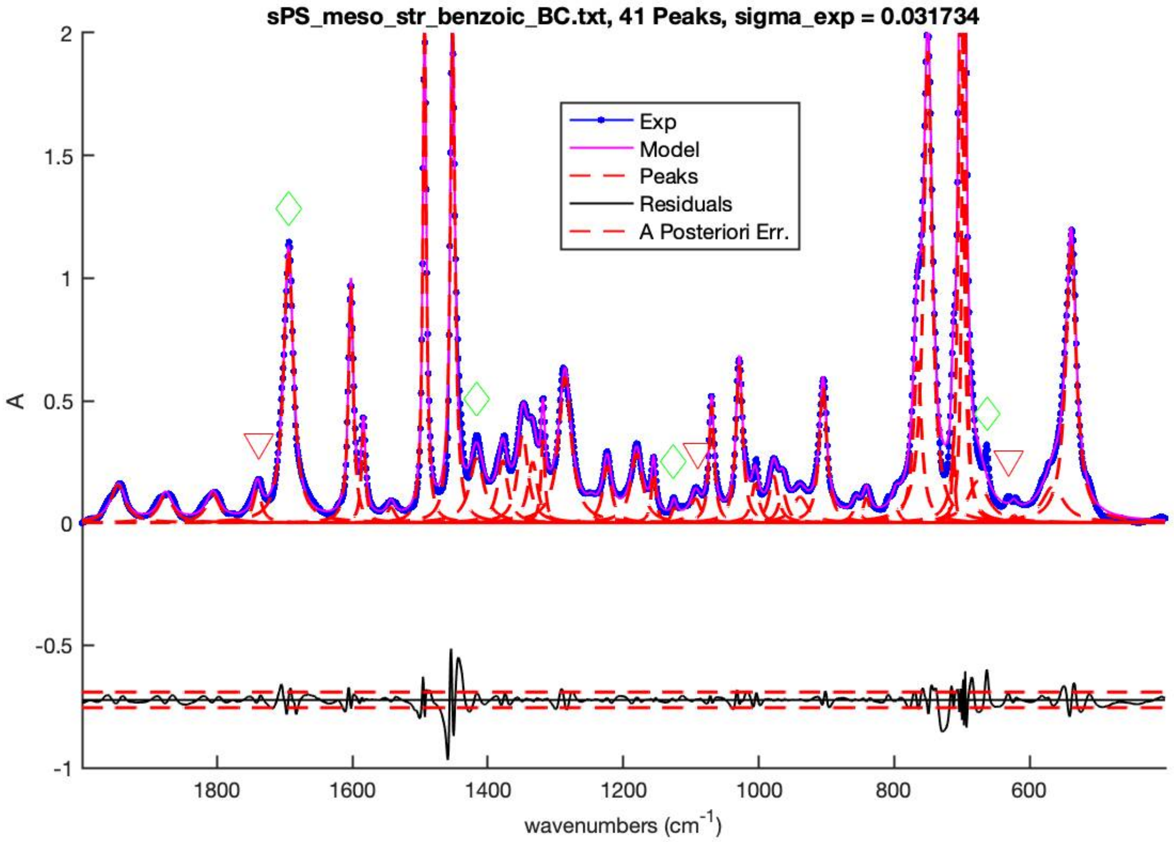

Figure S3. Portion of the spectrum of the sPS mesoform film, together with its fit in terms of sum of Lorentzians, the residuals on the bottom and the error levels. Peaks attributed to the monomer (dimer) of BA are marked with a red triangle (a green diamond).

Disclaimer/Publisher's Note: The statements, opinions and data contained in all publications are solely those of the individual author(s) and contributor(s) and not of MDPI and/or the editor(s). MDPI and/or the editor(s) disclaim responsibility for any injury to people or property resulting from any ideas, methods, instructions or products referred to in the content.
